# A Systematic Review Defining Early Anal Squamous Cell Carcinoma and Identifying Treatment

**DOI:** 10.3390/cancers17101646

**Published:** 2025-05-13

**Authors:** Cynthia Araradian, Mariah R. Erlick, Emmett Hunnicutt, J. Michael Berry-Lawhorn, Emily B. Rivet, Rebekka Duhen, Joseph Terlizzi, Tamzin Cuming, Sandy H. Fang

**Affiliations:** 1 Department of Surgery, Oregon Health & Science University, Portland, OR 97239, USA; erlick@ohsu.edu (M.R.E.); fangs@ohsu.edu (S.H.F.); 2 School of Medicine, Oregon Health & Science University, Portland, OR 97239, USA; hunnicue@ohsu.edu; 3 Division of Hematology Oncology, University of California San Francisco, San Francisco, CA 94115, USA; jmichael.berry@ucsf.edu; 4 Divisions of Colon and Rectal Surgery and Hospice and Palliative Medicine, Virginia Commonwealth University, Richmond, VA 23298, USA; emily.rivet@vcuhealth.org; 5 CEDAR (Cancer Early Detection and Advanced Research), Knight Cancer Institute, Oregon Health & Science University, Portland, OR 97239, USA; duhen@ohsu.edu; 6 Department of Surgery, Icahn School of Medicine at Mount Sinai, New York, NY 10011, USA; joseph.terlizzi@gmail.com; 7 Department of Surgery, Homerton University Hospital, London E9 6SR, UK; tamzin.cuming@nhs.net

**Keywords:** anal cancer, early anal cancer, SISCCA, squamous cell cancer, Nigro protocol, local excision

## Abstract

Background: Anal cancer screening has allowed for the diagnosis of early-stage anal cancers; however, guidelines for the treatment of early anal cancers remain mixed given the scarcity of clinical trials and data. Management ranges from chemoradiation therapy to local surgical excision. Methods: This paper’s objective is to clarify the definition of early anal cancer and its management. Approval for this systematic review was obtained through PROSPERO [CRD42022304327]. Three independent reviewers screened the studies and performed data analysis, with conflicts resolved by a fourth reviewer. All information is presented according to the Preferred Reporting Item for Systematic Reviews and Meta-Analysis (PRISMA). The Grades of Recommendation, Assessment, Development, and Evaluation (GRADE) criteria were used to assess the certainty of the evidence. Results: The MEDLINE, EMBASE and Cochrane databases were queried with 628 articles screened. A total of 15 articles were selected for inclusion. Early anal cancer was most often defined as T1-2N0M0 anal cancer but also included Superficially Invasive Squamous Cell Carcinoma (SISCCA). There were various treatments assessed for outcomes including local excision, chemoradiation, and radiation. The studies reported overall survival, cancer-specific survival, recurrence-free survival, and/or colostomy-free survival, leading to significant heterogeneity amongst the studies. No meta-analysis was possible. Conclusions: Poor-quality studies exist for the evaluation of the most effective treatment modality for early anal cancer. Surgical excision alone with adequate margins has equivocal results, but data is poor. Chemoradiation is successful but may be overtreatment. Randomized controlled studies are needed, as screening will result in earlier anal cancer diagnoses.

## Simple Summary

The prevalence of squamous cell carcinoma is increasing with the earlier detection of anal cancer, due in part to high-resolution anoscopy. Early anal cancer is poorly defined. The aim of this systematic review is to clarify the definition of early anal cancer and to identify the management of early anal cancer. The MEDLINE, EMBASE, and Cochrane databases were queried, and 15 articles met the inclusion criteria. The current treatments for early anal cancer include chemoradiation, radiation only, and local excision ± adjuvant therapy. The most common early anal cancer definition was T1-2N0M0. Overall, given that the studies vary in their primary outcomes and their conclusions, no meta-analysis was performed. The quality of existing research is considered low, and further high-quality studies are needed to identify the best treatment modality for early anal cancer.

## 1. Introduction

Anal cancer is a rare cancer of the gastrointestinal tract with a rapidly increasing incidence rate globally [1]. As the most common variant of anal cancer is squamous cell carcinoma (SCC), this review will focus on this subtype. SCC has a primary predisposing risk factor of infection with human papillomavirus (HPV), as well as factors increasing the likelihood of persistent HPV infection, including HIV status [2]. The high-risk HPV subtypes that are considered oncogenic include HPV 16, 18, 31, 33, 45, 52, and 58 [2]. Other risk factors include men who have sex with men, anoreceptive sex, gynecologic HPV disease, a history of solid organ transplant, and immunosuppression placing patients at risk of infection with other oncogenic viruses [3]. Treatment options and prognostication for anal cancer are significantly correlated with cancer stage. In anal cancer, as with many cancers, this is primarily determined by the TNM system. In short, the extent and size of the primary tumor, involvement of nearby lymph nodes, and metastatic spread to distant organs determine both the extent of treatment necessary as well as prognostication [4].

Screening of anal cancer can be completed with anal cytology and HPV genotyping in addition to high-resolution anoscopy (HRA). If anal cytology is abnormal, then HRA should be performed. Screening should include high-risk individuals including persons living with HIV (PLWHs) and men who have sex with men. The data have demonstrated that longitudinal follow-up of patients and surveillance play a role in the identification and management of cytological abnormalities, with counseling playing an instrumental role in compliance to routine screening [5]. Since the publication of the Anal HSIL/Cancer Outcomes Research (ANCHOR) trial in 2022, there has been a larger emphasis on the screening, identification, and treatment of not only high-grade squamous intraepithelial lesion (HSIL) but also anal cancer [6]. HRA was utilized to surveil HSIL in the ANCHOR study, and half of the cancers diagnosed in ANCHOR were T1N0. While there is a scarcity of HRA providers, data have shown that anal cancer screening and HSIL surveillance by HRA may lead to the detection of earlier-stage anal cancers. As the number of HRA providers increases, early-stage anal cancer detection rates will likely increase [7]. Given the closer monitoring with providers, it will also be of utmost importance to identify the effects of HPV vaccination in this high-risk population, with the goal of increased HPV clearance [5].

There is currently no defined classification of early anal cancer. Three stages are particularly important in understanding early anal cancer: superficially invasive squamous cell carcinomas (SISCCA) and T1/T2 node-negative disease. SISCCA is a unifying term for minimally invasive carcinoma in multiple anatomic sites including the genital tract (e.g., cervix, vulva, vagina, penis, scrotum) and anus. For anal cancer, SISCCA is defined as less than 3 mm of invasive depth from the basement membrane and 7 mm of horizontal spread, and it is amenable to complete excision as a form of conservative surgical management [8]. T1 anal cancers are <2 cm, while T2 anal cancers are ≥2 cm and <5 cm in size.

While SISCCA and T1-T2N0 lesions are well defined, there is no umbrella understanding—or even a consensus definition—of early anal cancer. Current recommendations focus on individual analysis of each patient, which is an important consideration in treatment selection but does not provide evidence-based outcomes data to guide shared decision-making [9]. Anal cancer staging itself is a work in flux: in 2023, anal cancer staging was restructured to better capture accurate nodal staging. As MRI technology has become more heavily utilized in cancer staging, there has been an increased occurrence of false positive nodal involvement, resulting in inaccurate upstaging of anal tumors [10]. This restructuring focuses on tumor size (T) versus nodal involvement (N) in light of the former’s more significant impact on overall survival [11].

Currently, there are no consensus recommendations on the treatment of early anal cancer. Treatment options include radiotherapy (RT), chemoradiation (CRT) with the use of the modified Nigro protocol, and local excision versus abdominoperineal resection (APR). In the 1970s, the Nigro protocol changed the standard of care for anal cancer from surgical management with an APR to a nonsurgical approach with the use of radiation and concurrent 5-fluorouracil (5-FU) with mitomycin C (MMC) [12,13]. It is well established that CRT is the treatment of choice for anal cancer; however, some of the seminal trials excluded patients with early-stage cancer [14,15,16,17,18,19,20]. While there is little doubt about the value of treating anal cancer including early-stage patients with CRT, there is a paucity of data suggesting that early anal cancer patients may be treated with local excision. The ideal excision margin is also currently unknown, with a discrepancy between 1 mm [21] and 1 cm perianus/2 mm anal canal margins [22]. Often, when the margin is unclear, patients are referred for subsequent CRT [23].

Given the knowledge gap, this systematic review aims to improve the diagnosis, prognostication, and treatment of early anal cancer with the following aims: (1) clarify the definition of early anal cancer; (2) evaluate the current literature that studies the management of early anal cancer.

## 2. Materials and Methods

This study was IRB-exempt. Approval for this systematic review was obtained through PROSPERO [CRD42022304327].

The literature search was conducted in accordance with the Preferred Reporting Items for Systematic reviews and Meta-Analysis (PRISMA) search strategy and screening protocol. Three databases were queried—EMBASE, MEDLINE, and Cochrane—using the complete search strategy (see Appendix A, Supplemental Text). In summary, the search terms focused on the early diagnosis, screening, staging and treatment of anal and perianal cancers. The search on MEDLINE was completed on 1 November 2024, on EMBASE 5 November 2024, and on Cochrane 11 December 2024.

The Covidence systematic review software (Veritas Health Innovation, Melbourne, Australia) was used to help streamline the screening process. Three independent reviewers completed the initial screening and full-text reviews. A fourth independent reviewer performed conflict resolution for the inclusion or exclusion of articles. Studies were included if they were retrospective or prospective cohort studies and randomized control trials that discussed patients with T1 and T2, stage I-II squamous cell anal cancer. Studies were excluded if they were expert opinions, other systematic reviews, case reports, case series, book chapters, letters to the editors, consensus guidelines, abstracts without a full-text component, and texts not translated into English. Studies were also excluded if they mentioned only advanced anal cancer diagnoses, including locally advanced and metastatic disease, and if other pathologic subtypes of anal cancer were discussed without mention of squamous cell carcinoma.

The data collected included patient demographics (age, gender, race), HIV and HPV status, treatment modalities, and results from each of the included texts. Given the heterogeneity of the papers in regard to the statistics and outcomes reported, we were unable to perform a meta-analysis after conferring with our statistician.

Certainty of evidence and risk of bias were assessed utilizing the Grading Recommendations, Assessment, Development and Evaluations (GRADE) framework. Three independent reviewers utilized the information from the studies to identify the certainty for each outcome.

## 3. Results

**Figure 1:**
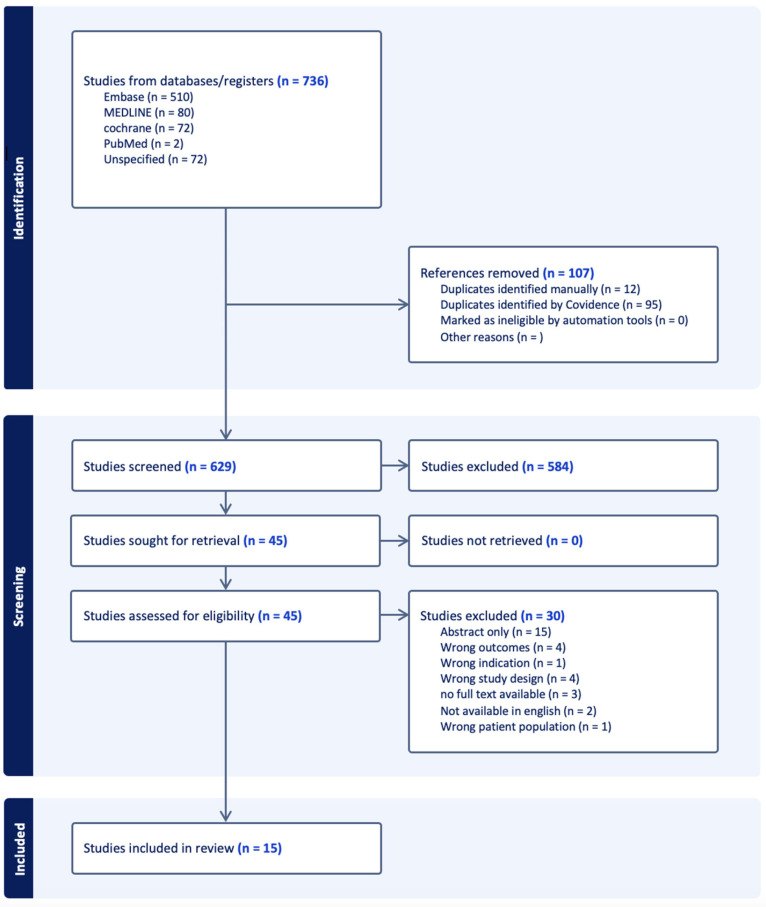
PRISMA flow diagram: The initial searched yielded 736 articles with 629 articles screened after the removal of duplicates. The initial screening consisted of article titles and abstracts. The second screening included full-text reviews. After full-text review, 15 articles were selected for the systematic review.

A total of 15 papers were included in this systematic review (Figure 1) [24,25,26,27,28,29,30,31,32,33,34,35,36,37,38]. The papers included patients with anal squamous cell cancer receiving different treatment interventions. Table 1 lists all 15 studies included in this systematic review. A total of 27,047 patients were included in the studies combined. Given the use of the same database, such as the National Cancer Database (NCDB), for multiple studies assessing the same time course, the same unidentified patient data may have been analyzed by multiple studies. Hence, the study patients may not represent unique patients. Also, given that two studies were included from Zilli et al., the patients in these studies were only counted once as the same patient dataset was used to assess similar outcomes [35,36].

Table 2 includes the patient demographics information extracted from each study. The majority of the included anal cancer study patients were white, female, and over the age of 50 years old. Many studies did not report the patients’ HIV and HPV status even though that may provide valuable information about the cohorts and their treatment outcomes.

### 3.1. Defining Early Anal Cancer

Ten out of the fifteen papers included in this review provided a definition for early anal cancer (Table 3). One of the papers, Alfa-Wali et al., defines early anal cancer as T1, node-negative disease [24]. Seven studies use T1-2, node-negative cancers as their definition of early anal cancer. Two studies utilized stages I-II as their definition of early anal cancer [28,38]. Of note, stage IIB includes T3 tumors or node positivity for smaller T1/T2 cancers. Two of the papers utilized SISCCA in their definition of anal cancer. Arana et al. had 7 of their 17 patients meet the diagnostic criteria for SISCCA, and Alfa-Wali et al. had 2 of their 15 patients meet the diagnostic criteria for SISCCA [24,25]. Maccabe et al. classified SISCCA as a sub-category of T1 tumors [37].

### 3.2. Chemoradiation vs. Radiation Alone

Three of the studies assessed chemoradiation with radiation alone, with two of them utilizing the NCDB data and one utilizing a retrospective cohort of patients from a single institution [29]. Table 4 summarizes the studies that compared chemoradiation with radiation therapy for early anal cancer management.

For both the Huffman et al. and Youssef et al. NCDB studies, chemoradiation (CRT) for 92% and 96% of the cohorts was compared to a minority treated with radiation (RT) alone for early anal cancers. Five-year overall survival (OS) was reduced with RT, at 65% and 79%, versus CRT, at 86% and 87%, respectively, with statistically significant differences [29,34]. The Youssef et al. paper differed in specifying younger patients (<70 years), and, in both studies, T2 patients were more likely to receive CRT. Characteristics in the Huffman study associated with patients who underwent radiation alone were age > 58 years, higher comorbidity scores, African American race, and having treatment at the start of the study period [29]. A challenge with the use of NCDB data is that the type of chemotherapy is not provided, so it is not known if consistent regimens were used across patients, whether there was a treatment break due to side effects, and HIV status.

Zilli et al. also evaluated the outcomes of early anal cancer patients treated with RT alone compared to CRT with the use of 5-FU and mitomycin [36] in a study involving much earlier patients, dating back to the 1970s. Twenty percent of their cohort were T1N0-staged and were more likely to be treated with radiation alone. Overall, 117 (80.1%) patients were diagnosed with T2N0M0 disease. A total of 75 (51%) patients received CRT, with a majority of these patients, 68, having T2 disease. One hundred twenty-two (84%) patients accomplished locoregional control (LRC). The LRC rates were 81.4% for the entire population, 92.9% for the T1 tumors, and 78.5% for the T2 tumors. Overall, the 5-year LRC rate for radiation alone was 75.5%, and for chemoradiation, it was 86.8% (*p* = 0.155). There was no overall difference between the treatment groups for colostomy-free survival [36].

### 3.3. Chemoradiation Alone

There were two retrospective reviews that looked at outcomes in patients with early anal cancers treated with chemoradiation. Table 5 summarizes the studies that utilized CRT only and their reported outcomes.

Bentzen et al. performed a 7-year retrospective review of 328 patients, of whom 142 (43%) had T1-2N0 disease [26]. However, the T1-2N0 disease outcomes were not reported separately, and local tumors were treated with different regimens. In the local tumor group, five (4%) patients underwent salvage surgery for residual tumor after the completion of CRT. Survival and LRC were not reported separately for early anal cancer [26]. In the local group, the residual tumor rate was low, and chemoradiation was effective.

Goksu et al. utilized NCDB to assess outcome disparities between African American and White patients with stage I-II ASCC treated with CRT from 2004 to 2016 [28]. Out of a total of 9311 patients, 8451 (90.8%) patients identified as White and 860 (9.2%) as African American. On multivariable analysis, African American patients had worse OS compared to White patients, with a 5-year OS of 71% vs. 75% (*p* = 0.007), and it took longer to initiate treatment for African American patients [28].

### 3.4. Different Chemotherapy Regimens

In a retrospective review of stage I-III anal cancer patients, Peixoto et al. discussed different chemotherapy (5-FU and mitomycin versus capecitabine and mitomycin) regimens with concurrent radiation (Table 6) [32]. As their cohort included patients from stages I-III, they did not specifically look at patients with T1/T2N0 cancers but did provide information regarding outcomes in this patient population. There was no significant difference in disease-free survival (DFS) and anal cancer-specific survival (ACSS) with the different chemotherapy regimens. The patient’s clinical T size (<5 cm or >5 cm), clinical N status, and HIV status were important prognostic factors for the DFS and ACSS [32]. Both chemotherapy regimens were equally efficacious.

### 3.5. Surgery with Chemoradiation

The Nigro protocol with concurrent chemoradiation has been the gold standard for anal cancer treatment, but quality-of-life studies have demonstrated a significant impact of the treatment on patients’ social and physical functions, including diarrhea and decreased sexual enjoyment [39]. Given the functional impairments for patients, there has been an interest in utilizing local excision for early anal cancer. The following two studies compare local excision with other regimens, including definitive or adjuvant CRT and abdominoperineal resection (APR), with results summarized in Table 7.

Suradkar et al. utilized the Surveillance, Epidemiology, and End Results (SEER) database (2004–2013) to evaluate 1530 patients with stage I-II anal cancer from a total of 3207 patients according to the treatment regimen as follows: radiation, local excision, APR, or neoadjuvant radiation with APR. Most of the patients underwent radiation 2772 (83.8%), 382 (11.6%) had a local excision, 77 (2.3%) underwent an APR, and 76 (2.3%) underwent neoadjuvant radiation followed by an APR. For the local excision group, although it is reported that 382 patients were in this group, there is missing staging information. Based on those that had staging reported, most patients who underwent local excision had stage I or II disease with well- or moderately differentiated tumors. The five-year OS rates for the radiation, local excision, APR, and neoadjuvant radiation with APR groups were 63.7%, 79.6%, 25.8%, and 41.8%, respectively (*p* < 0.001). When focusing specifically on radiotherapy and local excision, Cox regression analysis demonstrated that survival was similar between the groups when controlling for factors like age, gender, race, tumor size, and tumor differentiation. Patients who underwent APR or radiation followed by APR had overall worse outcomes, likely as a result of treatment failure [33]. Overall survival and cancer-specific survival were high for the local excision group. The SEER database has missing data and does not specify the location of the cancer within the anus (anal margin versus verge versus canal), nor does it specify measurements of the margins in local excision specimens. It also does not provide information on chemotherapy use. It is unknown how many of the patients in the radiation group received chemoradiation compared to radiation alone.

Berger et al. performed a 20-year single-institution retrospective review of 40 T1-2N0 anal cancer patients, of whom half the patients (11/20, 55% T1) underwent surgery followed by radiation, and the other half (5/20 25% T1) received definitive chemoradiation [27]. The groups were matched based on sex, age, tumor grade, and tumor location (anal canal vs. anal verge). Those who underwent surgery were incidentally diagnosed after the excision of what was thought to be a benign lesion and subsequently underwent adjuvant therapy. The adjuvant regimen was radiation only in 9 (45%) patients and chemoradiation in 11 (55%). One patient in the surgery first group had a local recurrence with a median 66-month follow-up. There was no significant difference in 5-year OS between the two groups, with a rate of 94.7% for surgery first and 86.5% for chemoradiation only (*p* = 0.36) [27]. Given that most patients in the surgery first group had T1 disease, the groups did not appear to be matched for tumor size.

### 3.6. Surgery Alone

There is currently no consensus on the appropriate margin size for local excision alone. The first three papers evaluate T1N0 and/or T2N0 anal cancer or SISCCA treated solely with local excision. The fourth paper evaluates T1-T3N0 and T1-T2N1 cancers. The results for the four studies are summarized in Table 8.

Alfa-Wali et al. evaluated 15 HIV-positive patients with T1 anal verge cancers managed with local excision with a margin of 1 cm or more [24]. The two patients who had SISCCA were included in the T1 cancer cohort. The 4-year median OS was 100% [23].

Arana et al. retrospectively analyzed 17 patients with T1 cancers, including SISCCA, who underwent local excision and matched these patients with 17 patients who had undergone excision for HSIL identified with the use of HRA, although it is not clear if HRA was used to guide SISCCA excision [25]. HIV-positive anal cancer patients in this cohort tended to be younger (43 years) than the HIV-negative patients (55 years). Local excision was defined as a margin of >2 mm for anal canal cancers and a margin of > 1 cm for anal margin cancers. If patients had positive margins, they underwent adjuvant CRT (12 patients, 70.6%). The 5-year OS was 100%. The cancer recurrence-free survival was 87% (95% CI 71–100%) at 5 years. Both patients that had recurrence had undergone adjuvant CRT and were salvaged with an APR. They also reported a 5-year HSIL recurrence-free survival of 58.8% but did not record ablation of background HSIL after cancer excision in the cancer group. Factors associated with an increased risk of HSIL recurrence in the cancer group were extensive anal disease, CD4 counts < 500, and lack of adjuvant radiation [25].

MacCabe et al. conducted a retrospective study that evaluated 39 patients with SISCCA and T1-T2N0 cancers who underwent local excision [37]. A total of 15 of the cancers were located in the anal canal and 24 at the perianal skin. Margins were considered adequate at 1 mm (United Kingdom and European guidelines). Positive margin (R1) resections occurred in 27 patients (69.2%), more frequently with anal canal cancers compared to perianal cancers (93.3% vs. 54.2%, *p* = 0.0006). Of the R1 resections, 9 patients underwent re-excision, and 20 patients had adjuvant CRT. The 5-year OS was 86.2% [95% CI 55.0–96.4] in anal canal cancers versus 95.7% in perianal cancers [95% CI 72.9–99.4], *p* = 0.607 [37].

A retrospective review by Brogden et al. evaluated 94 patients with stage I and II ASCC, of which 89 patients had T1-T2N0 early anal cancer [38]. Thirty-five patients (34 T1N0 and 1 T2N0) underwent local excision only. The authors concluded that patients with stage 1 cancer were less likely to receive chemoradiation when compared to stage 2 and more likely to have local excision of tumor alone as the only treatment for their malignancy. There was no reported statistically significant difference in 5-year disease-free survival and recurrence between treatment groups. They did report that eight patients with stage 1 disease had recurrence, and they found that those who had a local excision recurred later than those who received other treatment modalities [38].

### 3.7. Other

The remaining three articles did not fit into any of the above treatment regimen categories. The results are summarized in Table 9.

The Martin et al. paper was a survey-based study sent to 361 German institutions with radiation oncology departments [31]. A total of 84 (82.1%) institutions provided information specific to early anal cancer (T1-T2N0M0), with reported radiation doses of 54–59.4 Gy for early cancers, while 76 (75.2%) institutions used 59.4 Gy for locally advanced cancers [31].

Kabarriti et al. performed a retrospective review using NCDB with the inclusion of 5927 patients with nonmetastatic ASCC [30]. Patients were categorized as early anal cancer with T1-2N0M0 disease or advanced disease. In the early anal cancer patients, there was no difference in OS between HPV-positive and -negative disease with a 5-year OS of 79.3% for HPV-positive and 81.5% for HPV-negative patients [30].

Zilli et al. (2013) studied the treatment of inguinal node radiation therapy (INRT) with T2N0 disease [24]. They included 116 of their patients from their prior cohort, excluding the 30 T1N0 disease patients. The patients remained split into their initial treatment groups of chemoradiation or radiation only, but they also factored in INRT, which was used in 74 (63.8%) patients, although the patients chosen for this treatment were not described. Patients who received INRT were more likely to be in the CRT group (82%) compared to the RT-only group (17%). The 5-year locoregional control rate was 77.8% +/− 7.0% for patients without INRT and 80.1% +/− 5.0% for patients with INRT. Seven patients had an inguinal relapse during the 69-month follow-up period. Four patients treated with INRT had a groin relapse. The 5-year inguinal relapse-free survival was 93.3% +/− 3.2% with INRT and 90.4% +/− 5.4% without INRT [24]. Based on this study’s findings, there was no clear benefit to the elective use of INRT in patients. However, around 10% of patients with T1/T2 N0 disease experienced groin relapse [24].

### 3.8. GRADE Criteria

Given the heterogeneity amongst the treatment outcomes and overlapping NCDB data, there was a GRADE score of very low certainty for each of the treatment outcomes assessed by the studies in this paper (Table 10). The very low certainty was assigned given the differences in the outcomes reported, e.g., overall survival, recurrence-free survival, colostomy-free survival. The treatment group of different chemotherapy regimens was excluded from the GRADE criteria as it included just one paper.

## 4. Discussion

This review delineates the most commonly used definition of early anal cancer in mainly chemoradiation papers as T1-2N0M0 disease and SISCCA. This systematic review identified that treatment regimens have been disparate, and differing outcomes have been used, making a recommendation for the management of early anal cancer not possible. Chemoradiation was used in the majority of cases, with radiotherapy alone having worse outcomes even for early disease.

Other systematic reviews have looked at the treatment of early anal cancer and its management. Talwar et al. compared chemoradiation to radiation alone when looking at 5-year OS and 5-year disease-free survival (DFS) in stage 1 ASCC. They found that CRT resulted in a better OS but found no difference in DFS [9]. They emphasized that each patient should be evaluated on an individual level, and RT alone should be considered if the toxicities from chemotherapy are predicted to be unmanageable. They did not, however, include local excision as a comparator.

Werner et al. concluded that there is no difference in OS when comparing local excision with chemoradiation for the management of early-stage anal cancer, but they noted that more studies are needed on this comparison; the data are largely from older observational studies and pertained primarily to the anal canal rather than the perianus [40].

In brief, both reviews conclude that for most patients with early-stage ASCC, CRT with 5-FU and mitomycin remains the gold standard, provided it is tolerable and if local excision is not an option [9,40].

A systematic review by Spinelli et al. looked at perianal squamous cell carcinoma (pSCC) [29]. Research in this area has been limited due to the rarity of the condition, occurring much less frequently than anal canal cancer. Currently, National Comprehensive Cancer Network (NCCN) guidelines consider local excision for early-stage pSCC when appropriate surgical margins (≥1 cm) can be achieved with adequate oncologic resection and may not impact sphincter function [22]. However, European guidelines deem a 1 mm margin as acceptable for an adequate oncologic local excision [21].

Further research into pSCC is crucial for several reasons. Currently, NCCN guidelines have the same chemoradiation regimen for anal canal cancers and pSCC. However, differences in lymphatic drainage between the perianal and anal canal regions can influence the spread of cancer, which in turn can help determine the appropriate radiation field and treatment volume. Spinelli et al. suggested the possibility of modulating the elective RT volumes based on the T-stage of pSCC [41]. They suggested that T1 and small T2 lesions (<4 cm) could benefit from smaller treatment volumes to minimize morbidity and toxicity. They also recommended considering elective pelvic lymph node irradiation for larger T2N0 lesions (>4 cm), as this could decrease the chance of recurrence, which is more difficult to manage. However, the Zilli et al. paper, included in this review, indicated a 10% groin recurrence in T1-2N0 patients, and current regimens include routine pelvic lymph node irradiation [42].

In the upcoming randomized controlled trial of chemoradiation for T1-2N0 anal cancer (ACT 4), which has now closed to recruitment, no radiotherapy-alone arm was used, and comparison between 50.4 Gy and a reduced dose radiotherapy (41.4 Gy) was conducted with quality of life as an outcome measure [43].

Likewise, no variation in chemotherapy regimen was used in the PLATO series (ACT 3, 4, and 5) of trials. However, the question as to whether chemoradiation constitutes overtreatment for early anal cancers, particularly T1N0 and SISCCA, is worth considering. The data presented here are derived from low-volume retrospective observational studies. The ACT 3 study has recruited 90 patients and prospectively follows those with well-differentiated, perianal-only, fully excised T1N0 (only) cancers who did not receive CRT. European guidelines define adequate margins as 1 mm [21]. Cases of positive margins (<1 mm), poor differentiation, or lymphovascular or perineural invasion were treated with adjuvant CRT, as per the Berger study, and not with re-excision, which yielded poor outcomes in the Maccabe study [27,37,43].

Among the studies on local excision reported in this review, only one study followed patients with HRA. The results were variable, with good overall survival, but all studies had high positive margin rates. The studies reported have not addressed the question of whether 1 mm (European guidelines) [21] or 1 cm perianus/2 mm anal canal (United States guidelines) [22] is an adequate margin for excision alone.

The potential for overtreatment of early anal cancer must be weighed against the risks of positive margins and locoregional recurrence with excision. The upcoming results from the ACT 3 and ACT 4 trials will provide insight into the management of early anal cancer and better define the condition.

Capello et al. followed patients with HRA after chemoradiation (n = 30, T1-4 N0-1) or local excision (22 T1N0, 1 T2 N0) for anal cancer [7]. A total of 2 anal canal (AC) and 21 perianal (PA) lesions were locally excised compared to 18 AC and 12 PA in the CRT group. There was a high prevalence of HSIL detected in patients who underwent local excision only (17/23, 74%) versus chemoradiation (4/30, 13%) (*p* < 0.001). Background HSIL was ablated, and local recurrence was 0 in the 23 local excision patients, with 1 of 30 recurring in the CRT group; the recurrence was excised without salvage APR. This suggests that HRA may be helpful in finding early local recurrences of ASCC and that ablation of background HSIL may be the key to reducing local recurrence after all anal cancer treatments. Further prospective studies of HRA in the setting of anal cancer excision and postanal cancer treatment are required.

The main limitation of this review is that there is no high-quality data that helps draw conclusions about the best management of early anal cancer. The favorable outcomes with local excision point to its clinical utility for early anal cancer patients; however, a limitation of these four studies is the small sample size. Because of the small sample sizes, it is difficult to determine whether local excision alone is adequate for early anal cancer treatment, and this also raises the concern that we may be overtreating early-stage anal cancers. The ideal studies for assessing management would include randomized control trials and further prospective cohort studies that include larger numbers of patients, specifically looking at chemoradiation and local excision. The difficulty with the current literature is that many of the studies utilize databases like NCDB and SEER. These databases are helpful but lack several key pieces of information about patient management, including the chemotherapy regimen, immune competence, location of the cancer, and recurrence data. As multiple studies from NCDB are included with overlapping years with different inclusion/exclusion criteria, it becomes difficult to interpret the variance in the overall outcomes.

A key component to the management of early anal cancer is collecting data on outcomes internationally. The creation of the Multinational Anal Squamous Cell Carcinoma Registry and Audit (mASCARA) is a step towards collecting international data on patients with HSIL and anal cancer to better understand risk factors and optimal treatment strategies [44].

## 5. Conclusions

Anal cancer prevalence is increasing, and screening is being initiated, yet we do not have clear, evidence-based guidance on how to best treat patients with early anal cancer. Overall, seven of the studies included in this review define early anal cancer as T1-2N0 disease; however, excision-alone guidelines recommend excision only for T1N0 disease, so even this definition may change as the field develops. Chemoradiation and local excision demonstrated favorable outcomes in the studies presented. However, surveillance following local excision must be approached with caution. Hence, high-resolution anoscopy may be considered as a useful tool to identify and treat HSIL or early local recurrence within the anus. It will be important to see the results of the ACT 3 and 4 trials, future randomized control trials, and the use of international databases to determine how to generate the best oncologic outcomes for our patients.

## Figures and Tables

**Table 1:** Fifteen studies included in the systematic review: A brief overview of each of the 15 studies included in the review including year of publication, study design, years of inclusion, numbers of sites and patients, included treatments, and stages of disease. Abbreviations: SCC = squamous cell carcinoma, NCDB = National Cancer Database, EBRT = external beam radiation therapy, RT = radiation therapy, CRT = chemoradiation, CMT = chemotherapy, APR = abdominoperineal resection, SEER = Surveillance, Epidemiology, and End Results.

Author	Publication Year	Study Design	Years of Inclusion	Number of Institutions	Number of Participants	Anal Cancer Location and Stages	Treatments Studied
Alfa- Wali et al. [24]	2016	retrospective cohort	1986–2015	National Centre for HIV malignancies at Chelsea and Westminster Hospital in London	15	**anal verge**, T1N0M0	local excision only, surgical margin of >1 cm
Arana et al. [25]	2015	retrospective review	Jan 2007–December 2009	1—Diaconesses Hospital	17	T1N0M0- superficially invasive or invasive	local excision: margin of >2 mm for anal canal cancers and >1 cm for anal margin
Bentzen et al. [26]	2011	retrospective cohort	July 2000–June 2007	5—University Hospital of Northern Norway, Oslo University Hospital, Haukeland University Hospital, Olav University Hospital, University of Tromsø	328	nonmetastatic squamous cell carcinoma	definitive CRT
Berger et al. [27]	2012	retrospective cohort	1998–2008	1—University of Tuebin	40	T1-2N0 anal cancer	surgery with adjuvant CRT vs. primary chemoradiation
Goksu et al. [28]	2020	retrospective database (NCDB)	2004–2016	Database	9311	stage I-II squamous cell cancer	CRT
Huffman et al. [29]	2021	retrospective database (NCDB)	2004–2016	Database	2959	cT1N0M0 squamous cell of **anal canal**	EBRT with and without CMT
Kabarriti et al. [30]	2019	retrospective database (NCDB)	2008–2014	Database	5927	nonmetastatic squamous cell carcinoma	differences in radiation dose
Martin et al. [31]	2020	pattern of care survey		101 institutions (28% response rate—sent to 361)			
Piexoto et al. [32]	2016	retrospective review	1998–2013	British Columbia Cancer Agency	300	stages I-III SCC in 243/300 (81%)	CRT with 5-FU + mitomycin (FM) vs. capecitabine + mitomycin (CM)
Suradkar et al. [33]	2017	retrospective database (SEER)	2004–2013	Database	3307	**anal canal**	RT vs. local excision vs. APR vs. APR after RT
Youssef et al. [34]	2019	retrospective database (NCDB)	2004–2014	Database	4564	T1-2N0M0, 33.5% with T1 and 66.5% with T2	CRT vs. RT alone
Zilli et al. [35]	2013	retrospective cohort	1976–2008	1—Geneva University Hospital	116	T2N0M0	elective inguinal node radiation therapy in those treated with RT alone or CRT
Zilli et al. [36]	2011	retrospective cohort	1976–2008	1—Geneva University Hospital	146	T1-2N0M0 anal cancer, **anal canal and anal margin**	RT alone vs. CRT with 5-FU/ mitomycin
MacCabe et al. [37]	2020	retrospective cohort	2007–2019	1—University Hospitals Bristol and Weston	39	T1-T2N0M0, perianal and anal canal	local excision alone
Brogden et al. [38]	2021	retrospective cohort	2000–2020	1—NHS Foundation Trusts	94	T1-T3N0M0	CRT vs. RT vs. local excision alone vs. APR vs. defunctioning stoma

**Table 2:** Patient demographics: Patient demographic data obtained from each of the included papers. Given the variability in reported statistics, average age is reported as mean or median age. If the corresponding demographic data were not provided, NA is marked in the column representing not available. Abbreviations: APR = abdominoperineal resection, CRT = chemoradiation, RT = radiation therapy, CMT = chemotherapy, INRT = inguinal node radiation therapy.

Authors	Age	Sex	Race	HIV Positivity	HPV Positivity
Alfa-Wali et al. [24]	mean 49 years	NA	NA	15/15 (100%)	NA
Arana et al. [25]	median 47.7 years	female 5/17 (29.5%)	NA	10/17 (58.8%)	17/17 (100%)
Bentzen et al. [26]	median 63 years	NA	NA	6/328 (2.0%)	NA
Berger et al. [27]	median 58 years	female 25/40 (67.0%)	NA	NA	NA
Goksu et al. [28]	mean 58 years	female 6114/9311 (65.6%)	8451/9311 White (90.6%)	NA	NA
Huffman et al. [29]	median age 58	female 2019/2959 (68.2%)	2722/2959 (92.0%) White	NA	NA
Kabarriti et al. [30]	mean 57.3 for HPV tested group	female 3466/5927 (58.5%)	5002/5927 (85.3%) White	NA	3523/5927 (59.4%)
Martin et al. [31]	NA	NA	NA	NA	NA
Peixoto et al. [32]	median age 58	female 195/300 (65%)	NA	13/300 (4.0%)	NA
Suradkar et al. [33]	RT: mean 60 years Local excision:57 years APR: 64 years RT + APR: 56 years	RT: female 1884/2772 (68.0%) Local excision: female 192/382 (50.3%) APR: female 36/77 (46.8%) RT + APR: female 46/76 (60.5%)	RT: 86.8% White Local excision: 81.7% White APR: 88.3% White RT + APR: 93.4% White	NA	NA
Youssef et al. [34]	over 60 years old, 68.7% in CRT and 68.4% in RT	NA	White 90.5% in CRT group and 84.5% in RT	NA	NA
Zilli et al. (2013) [35]	median with INRT 65 years, without INRT 71 years	female 84/116 (72.4%)	NA	NA	NA
Zilli et al. (2011) [36]	median 66 years	female 105/146 (72.0%)	NA	NA	NA
Maccabe et al. [37]	median 62 years	female 29/42 (69.0%)	NA	NA	NA
Brogden et al. [38]	mean 53.1 years	female 16/94 (17.0%)	NA	57/94 (60.6%)	NA

**Table 3:** Definition of early anal cancer: The provided definitions for early anal cancer based on the 10 papers that defined it.

Author	Early Anal Cancer Definition
Alfa- Wali et al. [24]	T1N0M0
Berger et al. [27]	T1-2N0
Goksu et al. [28]	T1-T3N0, T1-T2N1M0
Kabarriti et al. [30]	T1-2N0
Martin et al. [31]	cT1-2N0M0
Youssef et al. [34]	cT1-2N0M0
Zilli et al. (2013) [35]	T1-2N0
Zilli et al. (2011) [36]	T1-2N0
Maccabe et al. [37]	T1-2N0
Brogden et al. [38]	T1-T3N0, T1-T2N1M0

**Table 4:** Studies comparing chemoradiation with radiation alone. The 3 studies included represent the studies comparing outcomes for patients with chemoradiation vs. radiation alone. The statistics provided include overall survival (OS), locoregional control (LRC), and colostomy-free survival (CFS). In the Zilli et al. study, the provided statistics include mean +/− standard deviation. Abbreviations: CRT = chemoradiation, RT = radiation.

Author	Number of Participants (Years of Study)	Anal Cancer Location and Stages Included	Treatments Studied	Overall Survival (OS)	Locoregional Control Rates (LRC)	Colostomy-Free Survival (CFS)
Huffman et al. [29]	2959 (2004–2016)	cT1N0M0 squamous cell of **anal canal**	CRT with and without CMT	5-year OS: 86% with CRT, 65% with RT only		
Youssef et al. [34]	4564 (2004–2014)	T1-2N0M0, 33.5% with T1 and 66.5% with T2	CRT vs. RT alone	5-year OS: 86.6% CRT and 79.1% RT, *p* = 0.114 T1 OS: 90.3% with CRT and 84.7% RT T2 OS: 84.7% CRT and 72.8% RT, *p* < 0.0001		
Zilli et al. [36]	146 (1976–2008)	T1-2N0M0 anal cancer	RT alone vs. CRT with 5-FU/mitomycin Median follow-up 62.5 months	Estimated 5-year actuarial OS for all patients: 75.4% +/− 3.9%	5-year LRC for RT alone: 75.5% +/− 6% 5 year LRC for CRT: 88.5% +/− 4.5% 5-year LRC for T1 tumors: 92.9 ± 6.9% 5-year LRC for T2 tumors: 78.5 ± 4.1%	Entire cohort: 82.4% +/− 3.5% RT only: 79.9% +/− 5.6% CRT only: 84.6% +/− 4.5%, *p* = 0.567

**Table 5:** Chemoradiation only studies: Two studies assessed the outcomes using chemoradiation only. The statistics included overall survival (OS), cancer-specific survival (CSS), and recurrence-free survival (RFS). Abbreviations: ASCC = anal squamous cell carcinoma, CRT = chemoradiation, RT = radiation, EBRT = external beam radiation therapy.

Author	Number of Participants	Anal Cancer Location and Stages Included	Treatments Studied	Treatment Sequence	Relapses	Overall Survival (OS)	Cancer-Specific Survival (CSS)	Recurrence-Free Survival (RFS)
Bentzen et al. [26]	328	nonmetastatic squamous cell carcinoma	CRT only with RT (EBRT)	T1-T2N0 tumors: EBRT with 5-FU and mitomycin T3-T4 or N1 + 2: courses of neoadjuvant chemo with cisplatin and 5FU and concurrent CRT with EBRT	recurrence: 73/328 (24%), recurrence treated with surgical resection in 32, palliative intent in 40 patients	3-year OS = 79% 5-year OS = 66%	3-year CSS = 84% 5-year CSS = 75%	3-year RFS = 79% 5-year RFS = 74%
Goksu et al. [28]	9311	stage I–II ASCC	CRT only			5-year OS for African American patients: 71% vs. White 75%, *p* < 0.001		

**Table 6:** Different chemotherapy regimens: There was only one study that addressed different chemotherapy regimens and specifically looked at cancer-specific survival (CSS) and disease-free survival (DFS). Abbreviations: CM = capecitabine and mitomycin, FM = 5-fluorouracil and mitomycin.

Author	Number of Participants	Anal Cancer Location and Stages Included	Treatments Studied	Results	Cancer-Specific Survival (CSS)	Disease-Free Survival (DFS)
Peixoto et al. [32]	300	stages I–III ASCC	chemoradiation with 5-FU + mitomycin (FM) (194 patients, 64.6%) vs. capecitabine + mitomycin (CM) (106 patients, 35.3%)	only HIV status, clinical T size, and clinical N status were prognostic factors for DFS and ACSS	2-year anal cancer-specific survival: 88.7% CM (95% CI [81.8–95.5%]) and 87.5% (95%CI 82.8–92.2]) FM *p* = 0.839	79.7% (95% CI [71.7–88.3]) for CM and 78.8% (95%CI [73–84.6%]) for FM *p* = 0.663

**Table 7:** Chemoradiation vs. surgery: Two of the included studies assessed outcomes with chemoradiation vs. surgery of local excision. The two studies had outcomes of overall survival (OS), cancer-specific survival (CSS), locoregional control rates (LRC), and colostomy-free survival (CFS). Abbreviations: CRT = chemoradiation, RT = radiation, APR = abdominoperineal resection.

Author	Number of Participants	Anal Cancer Location and Stages Included	Treatments Studied	Overall Survival (OS)	Cancer-Specific Survival (CSS)	Locoregional Control Rates (LRC)	Colostomy-Free Survival (CFS)
Berger et al. [27]	40	T1-2N0 anal cancer	adjuvant CRT vs. primary CRT	5-year OS: 90% 10-year OS: 84.7% 5-year OS: surgery then adjuvant CRT group 94.7% 5-year OS: CRT group 86.5 *p* = 0.36		for surgery with adjuvant CRT-95% CRT-100% *p* = 0.32	5-year CFS 95% in both groups
Suradkar et al. [33]	3307	anal canal; all stages included 1530 patients with stage I and II anal cancer	RT vs. local excision vs. APR vs. APR after RT	5-year OS: radiation: 63.7% local excision: 79.6% APR: 28.5% RT + APR: 41.8% *p* < 0.001	RT: 79.6% local excision: 92.5% APR: 75.6% RT + APR: 58.8%, *p* < 0.001		

**Table 8:** Surgery alone: Four of the included papers assessed outcomes with local excision alone. They report overall survival and recurrence-free survival and discuss relapses. Abbreviations: EBRT = external beam radiation therapy, CRT = chemoradiation, RT = radiation, APR = abdominoperineal resection.

Author	Number of Participants	Anal Cancer Location and Stages Included	Treatments Studied	Treatment Sequence	Relapses	Overall Survival (OS)	Recurrence-Free Survival (RFS)
Alfa-Wali et al. [24]	15	**Anal verge**, T1N0M0	Local excision only, with a surgical margin of >1 cm			5-year OS 100%	
Arana et al. [25]	17	T1N0M0, superficially invasive or invasive	Local excision: margin of >2 mm for anal canal cancers, >1 cm for anal margin with or without adjuvant EBRT	Local excision, followed by EBRT if margins are inadequate (EBRT performed in 12/17)	2/17 (11.7%) had local recurrence, salvaged by APR	5-year OS: 100%	Cancer RFS 5-year: 87% HSIL RFS 5-year: 58.8%
MacCabe et al. [37]	39	T1-T2N0M0	Local excision only (margin > 1 mm)	Local excision followed by re-excision vs. adjuvant therapy	R1 excisions: T1 = 15/24 (62.5%), T2 = 12/14 (85.7%)	5-year OS: 86.2% [95% CI 55.0–96.4] anal canal; 95.7% [95% CI 72.9–99.4] perianal, *p* = 0.607	
Brogden et al. [38]	94	T1-T3N0M0, T1-T2N1M0	CRT vs. RT only vs. local excision of tumor with other treatment modality vs. APR vs. defunctioning stoma vs. local excision of tumor only	No margin size specified for local excision	3 (9%) had recurrence in the local excision-only group	No difference in 5-year disease-free survival	

**Table 9:** Other studies: The remaining three studies reported different treatment regimens or outcomes. In the study by Zilli et al., the results are reported as mean +/− standard deviation. Abbreviations: IMRT = intensity-modulated radiotherapy, ASCC = anal squamous cell carcinoma, RT = radiation therapy, INRT = inguinal node radiation therapy, CRT = chemoradiation.

Author	Number of Participants	Anal Cancer Location and Stages Included	Treatments Studied	Relapses	Results	Overall Survival (OS)	Locoregional Control Rates (LRC)	Colostomy-Free Survival	Inguinal Relapse-Free Survival
Kabarriti et al. [30]	5927	nonmetastatic ASCC	HPV-positive vs. HPV-negative disease; differences in radiation dose		No difference in OS for early anal cancer for HPV-negative vs. HPV-positive patients	Entire cohort: 3-year 78.1% 5-year 69.9% 5-year OS for HPV+ 79.3% and HPV− 81.5%			
Martin et al. [31]	101 institutions; 45 (44.6%) outpatient radiation oncology centers; 20 (19.8%) university affiliated departments; 36 (35.6%) non-university clinics				Most institutions use IMRT; 94% use 5-FU/mitomycin-based CRT				
Zilli et al. (2013) [35]	116	T2N0M0	Elective inguinal node radiation therapy in those treated with RT alone or CRT	22 patients with recurrence; 7 patients with inguinal relapse	Patients with INRT were more likely to be treated with CRT rather than RT alone		With INRT: 5-year 84.5% +/− 5.8% 5 year without INRT: 77.8% +/− 7% RT only: 71% +/− 7.2% CRT: 85.4% +/− 4.5%	Entire cohort: 79.4% +/− 4.2% For RT group: 74.2% CRT group: 83%	Entire cohort: 5-year 92.3% With INRT: 93.3%+/− 3.2% Without INRT: 90.4% +/− 5.4%, *p* = 0.733

**Table 10:** GRADE criteria: The GRADE criteria for each of the treatment outcomes. Very low certainty reported for each of the outcomes.

Certainty Assessment							Certainty *
№ of Studies	Study Design	Risk of Bias	Inconsistency	Indirectness	Imprecision	Other Considerations	
**Chemoradiation vs. Radiation**							
3	Non-randomized studies	Serious	Serious	Serious	Serious	None	⨁◯◯◯ Very low
**Chemoradiation Alone**							
2	Non-randomized studies	Serious	Very Serious	Serious	Serious	None	⨁◯◯◯ Very low
**Surgery with Chemoradiation**							
2	Non-randomized studies	Serious	Serious	Serious	Serious	None	⨁◯◯◯ Very low
**Surgery Alone**							
4	Non-randomized studies	Serious	Not Serious	Not Serious	Serious	None	⨁◯◯◯ Very low

* GRADE Working Group grades of evidence; High certainty: we are very confident that the true effect lies close to that of the estimate of the effect. Moderate certainty: we are moderately confident in the effect estimate: the true effect is likely to be close to the estimate of the effect, but there is a possibility that it is substantially different. Low certainty: our confidence in the effect estimate is limited: the true effect may be substantially different from the estimate of the effect. Very low certainty: we have very little confidence in the effect estimate: the true effect is likely to be substantially different from the estimate of effect.

## Abbreviations

The following abbreviations are used in this manuscript:

PRISMA	Preferred Reporting Item for Systematic Reviews and Meta-Analysis
GRADE	Grades of Recommendation, Assessment, Development, and Evaluation
SISCCA	Superficially Invasive Squamous Cell Carcinoma
SCC	squamous cell carcinoma
HPV	human papilloma virus
ANCHOR	Anal HSIL/Cancer Outcomes Research
HSIL	high-grade squamous intraepithelial lesion
HRA	high-resolution anoscopy
T	stage, tumor size
N	stage, nodal involvement
RT	radiation therapy, radiotherapy
CRT	chemoradiation
APR	abdominoperineal resection
5-FU	5-fluorouracil
MMC	mitomycin C
NCDB	National Cancer Database
FM	5-FU + mitomycin
CM	capecitabine + mitomycin
SEER	Surveillance, Epidemiology, and End Results
OS	overall survival
LRC	locoregional control rates
CFS	colostomy-free survival
CSS	cancer-specific survival
RFS	recurrence-free survival
EBRT	external beam radiation therapy
DFS	disease-free survival
ACSS	anal cancer-specific survival
R	residual tumor classification
INRT	inguinal node radiation therapy
pSCC	perianal squamous cell carcinoma
NCCN	National Comprehensive Cancer Network
AC	anal canal
PA	perianal
mASCARA	Multinational Anal Squamous Cell Carcinoma Registry and Audit

## Appendix A. Supplemental Text

Full search strategy:
((‘early diagnosis’/exp OR ‘mass screening’/exp OR (((early OR earlier OR earliest OR routin*) NEAR/3 (screen* OR detect* OR diagnos* OR discover* OR stag*)) OR (mass NEAR/3 screen*))) AND ((((((anus* OR anal OR anally OR anorect* OR perianal* OR perianus) NEAR/5 (cancer* OR neoplas* OR tumo$r* OR malig* OR carcino* OR adenocarcin* OR melanom* OR verge)) OR siscca) AND (anus* OR anal OR anally OR anorect* OR perianal* OR perianus OR cancer* OR neoplas* OR tumo$r* OR malig* OR carcino* OR adenocarcin* OR melanom* OR verge OR siscca) NEAR/5 (early OR earlier OR stag* OR detect* OR microinvas* OR invas* OR ‘excision only’ OR ‘only excision’ OR ‘excision alone’ OR t1 OR ‘carcinoma in situ’ OR tis OR ‘stage i’ OR ‘stage i?’ OR ‘stage 1’ OR ‘stage 1?’ OR ‘stage one’)) AND (treat* OR therap* OR pharmacother* OR chemother* OR chemoradiat* OR surg* OR excis* OR radiother* OR manag* OR interven* OR protocol*)) OR ((((((anus* OR anal OR anally OR anorect* OR perianal* OR perianus) NEAR/5 (cancer* OR neoplas* OR tumo$r* OR malig* OR carcino* OR adenocarcin* OR melanom* OR verge)) OR siscca) AND (anus* OR anal OR anally OR anorect* OR perianal* OR perianus OR cancer* OR neoplas* OR tumo$r* OR malig* OR carcino* OR adenocarcin* OR melanom* OR verge OR siscca) NEAR/5 (early OR earlier OR stag* OR detect* OR microinvas* OR invas* OR ‘excision only’ OR ‘only excision’ OR ‘excision alone’ OR t1 OR ‘carcinoma in situ’ OR tis OR ‘stage i’ OR ‘stage i?’ OR ‘stage 1’ OR ‘stage 1?’ OR ‘stage one’)) AND (treat* OR therap* OR pharmacother* OR chemother* OR chemoradiat* OR surg* OR excis* OR radiother* OR manag* OR interven* OR protocol*)) AND (treat* OR therap* OR pharmacother* OR chemother* OR chemoradiat* OR surg* OR excis* OR radiother* OR manag* OR interven* OR protocol) NEAR/5 (anus* OR anal OR anally OR anorect* OR perianal* OR perianus))) AND [embase]/lim) NOT ((‘early diagnosis’/exp OR ‘mass screening’/exp OR (((early OR earlier OR earliest OR routin*) NEAR/3 (screen* OR detect* OR diagnos* OR discover* OR stag*)) OR (mass NEAR/3 screen*))) AND ((((((anus* OR anal OR anally OR anorect* OR perianal* OR perianus) NEAR/5 (cancer* OR neoplas* OR tumo$r* OR malig* OR carcino* OR adenocarcin* OR melanom* OR verge)) OR siscca) AND (anus* OR anal OR anally OR anorect* OR perianal* OR perianus OR cancer* OR neoplas* OR tumo$r* OR malig* OR carcino* OR adenocarcin* OR melanom* OR verge OR siscca) NEAR/5 (early OR earlier OR stag* OR detect* OR microinvas* OR invas* OR ‘excision only’ OR ‘only excision’ OR ‘excision alone’ OR t1 OR ‘carcinoma in situ’ OR tis OR ‘stage i’ OR ‘stage i?’ OR ‘stage 1’ OR ‘stage 1?’ OR ‘stage one’)) AND (treat* OR therap* OR pharmacother* OR chemother* OR chemoradiat* OR surg* OR excis* OR radiother* OR manag* OR interven* OR protocol*)) OR ((((((anus* OR anal OR anally OR anorect* OR perianal* OR perianus) NEAR/5 (cancer* OR neoplas* OR tumo$r* OR malig* OR carcino* OR adenocarcin* OR melanom* OR verge)) OR siscca) AND (anus* OR anal OR anally OR anorect* OR perianal* OR perianus OR cancer* OR neoplas* OR tumo$r* OR malig* OR carcino* OR adenocarcin* OR melanom* OR verge OR siscca) NEAR/5 (early OR earlier OR stag* OR detect* OR microinvas* OR invas* OR ‘excision only’ OR ‘only excision’ OR ‘excision alone’ OR t1 OR ‘carcinoma in situ’ OR tis OR ‘stage i’ OR ‘stage i?’ OR ‘stage 1’ OR ‘stage 1?’ OR ‘stage one’)) AND (treat* OR therap* OR pharmacother* OR chemother* OR chemoradiat* OR surg* OR excis* OR radiother* OR manag* OR interven* OR protocol*)) AND (treat* OR therap* OR pharmacother* OR chemother* OR chemoradiat* OR surg* OR excis* OR radiother* OR manag* OR interven* OR protocol) NEAR/5 (anus* OR anal OR anally OR anorect* OR perianal* OR perianus))) AND [medline]/lim).
